# Clinical study on the value of TyG index combined with systemic immune-inflammation index for screening hospitalized patients with type 2 diabetic kidney disease

**DOI:** 10.3389/fendo.2026.1854268

**Published:** 2026-05-28

**Authors:** Qiuyun Song, Guangzhi Yang, Chen Sun, Xiaolong Chen

**Affiliations:** Department of Clinical Laboratory,Yancheng Third People’s Hospital, The Yancheng School of Clinical Medicine of Nanjing Medical University, Yancheng, Jiangsu, China

**Keywords:** predictive value, risk stratification, systemic immune-inflammation index, TyG index, type 2 diabetic kidney disease

## Abstract

**Objective:**

To evaluate the screening value of the triglyceride-glucose index (TyG) combined with the systemic immune-inflammation index (SII) for diabetic kidney disease (DKD) in hospitalized patients with type 2 diabetes mellitus (T2DM).

**Methods:**

A retrospective analysis was conducted on 335 hospitalized T2DM patients (175 with DKD, 160 without DKD) at Yancheng Third People’s Hospital from 2022 to 2024. DKD was defined according to KDIGO 2022 criteria persistent urinary albumin-to-creatinine ratio (UACR) ≥30 mg/g and/or estimated glomerular filtration rate (eGFR) <60 mL/min/1.73m² lasting for >3 months. Clinical data were collected, and the TyG index and SII were calculated. Due to severe skewness, SII was log10-transformed (lgSII). Logistic regression was used to construct five progressive prediction models. Model performance was evaluated using the area under the receiver operating characteristic curve (AUC), DeLong’s test, calibration curves, and decision curve analysis (DCA). Internal validation was performed via Bootstrap (2000 repetitions) and 5-fold cross-validation.

**Results:**

TyG and lgSII were significantly higher in the DKD group compared to the non-DKD group (P<0.001). The final streamlined model (including TyG, lgSII, diabetes duration, systolic blood pressure, and HbA1c) achieved an AUC of 0.850. The Hosmer-Lemeshow test indicated good calibration (P = 0.257). The optimal cut-off values were TyG >10.03 and lgSII >2.76, yielding a sensitivity of 74.3% and a specificity of 85.0%. The high-risk group (both indicators above cut-offs) had a DKD prevalence of 96.8%. Bootstrap validation yielded a mean AUC of 0.855, indicating robust model stability.

**Conclusion:**

The combination of TyG and SII demonstrates favorable screening efficacy and model stability for identifying DKD among hospitalized T2DM patients. This model provides preliminary evidence for a low-cost, readily available tool for rapid risk stratification, but requires external validation before clinical implementation.

## 1 Introduction

Diabetic kidney disease (DKD) stands as one of the most critical microvascular complications of type 2 diabetes mellitus (T2DM) and has emerged as the foremost cause of end-stage renal disease (ESRD) worldwide (1, 2). Epidemiological studies indicate that approximately 20%–40% of T2DM patients will eventually develop DKD, imposing not only profound impairments on quality of life but also a substantial socioeconomic burden (3). Early-stage DKD is often clinically silent, by the time overt proteinuria or a progressive decline in renal function becomes apparent, the underlying pathological alterations are frequently irreversible (4). Consequently, early detection and timely intervention are pivotal to slowing disease progression and improving long-term patient outcomes.

Current clinical guidelines recommend screening for DKD primarily based on the urinary albumin-to-creatinine ratio (UACR) and estimated glomerular filtration rate (eGFR) (5, 6). However, both metrics exhibit notable limitations: UACR can be influenced by various transient factors, including acute infections, physical exertion, and urinary tract abnormalities, leading to considerable variability. Meanwhile, eGFR may lack sufficient sensitivity in early renal impairment, often remaining within the normal range despite incipient functional decline, thus resulting in diagnostic oversight (7). Therefore, the identification of reliable, cost-effective, and readily obtainable biomarkers or their combinations represents a pressing research priority to enhance early DKD detection.

Recent research highlights the central roles of insulin resistance and chronic low-grade inflammation in the pathogenesis of DKD. The triglyceride-glucose (TyG) index, derived from fasting triglyceride and glucose levels, serves as a simple and reliable surrogate marker of insulin resistance (8, 9). Elevated TyG has been associated with microalbuminuria and reduced eGFR in T2DM patients. The systemic immune-inflammation index (SII) is a novel inflammatory marker derived from routine blood counts (10, 11). It integrates parameters of innate immunity and adaptive immunity, dynamically reflecting the body’s immune-inflammatory balance. Chronic low-grade inflammation is considered a core mechanism linking metabolic dysregulation to renal structural and functional damage in DKD pathogenesis, promoting glomerulosclerosis and renal interstitial fibrosis (12).

Although prior investigations have separately examined the relationships of TyG or SII with DKD, most studies have been constrained by a univariate focus, limited sample sizes, and insufficient evaluation of their combined discriminative performance within a well-defined hospitalized T2DM cohort (13). Moreover, the translation of these two indices, representing distinct yet interconnected “metabolic” and “inflammatory” axes into a practical, clinically applicable risk-stratification framework remains largely unexplored (14). Notably, both TyG and SII can be derived from routinely available laboratory data without incurring additional costs, offering the advantages of accessibility, reproducibility, and non-invasiveness, thereby positioning them as promising candidates for rapid DKD risk assessment in hospitalized T2DM patients.

This study aims to conduct a retrospective analysis to systematically develop and validate DKD prediction model that integrates the TyG index and SII. We will evaluate the screening performance of this combined model in a cohort of hospitalized T2DM patients and rigorously assess its discriminative capacity, calibration, and clinical utility through internal validation and decision-curve analysis. Ultimately, we seek to establish a low-cost, readily implementable risk-stratification tool capable of facilitating early identification of high-risk individuals and optimizing clinical resource allocation.

## 2 Materials and methods

### 2.1 Study participants

A total of 335 T2DM patients hospitalized in the Department of Endocrinology at Yancheng Third People’s Hospital between January 2022 and December 2024 were consecutively enrolled via the hospital’s Laboratory Information System (LIS). Patients were categorized into a DKD group (n=175) and a non-DKD group (n=160).

Inclusion Criteria: (1) Diagnosis of T2DM based on American Diabetes Association criteria (HbA1c ≥6.5%, fasting plasma glucose ≥7.0 mmol/L, or 2-hour post-load glucose ≥11.1 mmol/L). (2) Diagnosis of DKD based on the KDIGO 2022 Clinical Practice Guideline for Diabetes Management in CKD: persistent UACR ≥30 mg/g (i.e., A2 or A3) and/or persistent eGFR <60 mL/min/1.73m² (i.e., G3a-G5) in the absence of other primary kidney diseases. Diabetic retinopathy, when present, served as supportive evidence but was not mandatory for the diagnosis. (3) Complete clinical data; (4) Age ≥18 years.

Exclusion Criteria: (1) Type 1 diabetes or other specific types of diabetes; (2) Acute infection, severe hepatic or renal dysfunction (non-diabetic origin), malignancy, hematological diseases, or autoimmune disorders; (3) Recent use of corticosteroids or immunosuppressants; (4) Pregnancy or lactation.

This study was approved by the Ethics Committee of Yancheng Third People’s Hospital (Approval No.: Ethics-2025-107). Due to the retrospective nature of the study, the requirement for informed consent was waived. All personal identifying information was anonymized prior to analysis.

### 2.2 Data collection and laboratory measurements

Clinical and laboratory data were collected within 24 hours of admission. General information included gender, age, diabetes duration, body mass index (BMI), systolic blood pressure (SBP) hospitalization days, smoking history, alcohol history, and hypertension history. Hypoglycemic treatment regimens were extracted from admission records and classified into four categories: no medication/dietary control, oral antidiabetic agents only, insulin only, and oral agents combined with insulin. Comorbidities recorded included coronary heart disease, cerebrovascular disease, peripheral vascular disease, diabetic retinopathy, and diabetic peripheral neuropathy. Laboratory indicators included fasting plasma glucose (FPG), glycated hemoglobin (HbA1c), triglyceride (TG), total cholesterol (TC), high-density lipoprotein cholesterol (HDL-C), low-density lipoprotein cholesterol (LDL-C), blood urea nitrogen (BUN), serum creatinine (Scr), albumin (ALB), alanine aminotransferase (ALT), aspartate aminotransferase (AST), neutrophil count (N), lymphocyte count (L), and platelet count (PLT).

All laboratory tests were performed in the hospital’s central laboratory, which is certified under ISO 15189. Biochemical parameters were measured using a Roche Cobas 8000 analyzer. Complete blood counts were obtained using a Sysmex XN-2000 hematology analyzer. HbA1c was measured using a Medconn PT 6000 analyzer. All procedures followed standardized protocols with strict internal and external quality control.

### 2.3 Calculation of TyG and SII

TyG Index: TyG = Ln [TG (mg/dL) × FPG (mg/dL)/2]. Since TG and FPG were measured in mmol/L in this study, conversion was applied: FPG (mg/dL) = FPG (mmol/L) × 18; TG (mg/dL) = TG (mmol/L) × 88.57.

Systemic Immune-Inflammation Index (SII): SII = PLT (×10^9^/L) × N (×10^9^/L)/L (×10^9^/L).

Due to a severely right-skewed distribution, SII was log10-transformed for analysis and denoted as lgSII. This transformation normalizes the data for parametric analyses and ensures that the derived optimal cut-off values for SII are based on a more stable and interpretable scale.

### 2.4 Statistical analysis

Statistical analyses were performed using Python 3.11. Continuous variables were tested for normality using the Shapiro-Wilk test. Normally distributed data are presented as mean ± standard deviation and compared using Student’s t-test, with effect size expressed as Cohen’s d. Non-normally distributed data are presented as median (interquartile range) and compared using the Mann-Whitney U test, with effect size as Cliff’s delta. Categorical variables are presented as frequencies (percentages) and compared using the chi-square test or Fisher’s exact test. Spearman’s rank correlation coefficient (ρ) was used to assess correlations between variables.

### 2.5 Model development and validation

Five logistic regression models were constructed: Univariate model with TyG, Univariate model with lgSII, Bivariate model with TyG + lgSII, Full model incorporating TyG, lgSII, and clinically relevant covariates, Streamlined model derived from Model 4 using backward stepwise selection. Model performance was evaluated by the area under the ROC curve (AUC). AUCs were compared using DeLong’s test. Model calibration was assessed using the Hosmer-Lemeshow goodness-of-fit test and calibration plots. Clinical utility was evaluated via decision curve analysis (DCA) across a threshold probability range of 10%-40%. Internal validation was performed using Bootstrap resampling (2000 repetitions) and 5-fold cross-validation (20 repetitions).

### 2.6 Risk stratification

Optimal cut-off values for TyG and lgSII in the streamlined model were determined by maximizing Youden’s index from the ROC analysis. Patients were stratified into three risk groups: Low-risk (both TyG and lgSII below cut-offs), Medium-risk (either TyG or lgSII above its cut-off), and High-risk (both TyG and lgSII above cut-offs). The prevalence of DKD and corresponding ORs were calculated for each stratum.

## 3 Results

### 3.1 Baseline characteristics

Baseline characteristics are summarized in **Table 1**. The DKD group had significantly higher TyG (9.79 ± 0.87 vs. 9.08 ± 0.74, P<0.001, Cohen’s d=0.875) and lgSII (2.70 ± 0.26 vs. 2.49 ± 0.22, P<0.001, Cohen’s d=0.868) compared to the non-DKD group. The DKD group also had a longer diabetes duration, higher systolic blood pressure, and higher HbA1c levels (all P<0.05). No significant differences were found in age, gender, BMI, smoking, or alcohol history between the two groups. Detailed comparisons of diabetes duration strata, hypoglycemic regimens, hospitalization days, and macrovascular/microvascular complications are presented in **Table 1**.

**Table 1:** Baseline characteristics of the study population.

Variable	Non-DKD group (n=160)	DKD group (n=175)	Test statistic	P-value	Effect size
Core indicators					
TyG	9.08 ± 0.74	9.79 ± 0.87	-8.002	<0.001	0.875^‡^
SII	288.78 (225.89, 453.66)	528.44 (336.29, 715.94)	7392	<0.001	0.472^Δ^
lgSII	2.49 ± 0.22	2.70 ± 0.26	-7.938	<0.001	0.868^‡^
Demographic characteristics					
Age	59.53 ± 11.44	61.23 ± 12.46	-1.294	0.196	0.142^‡^
Gender (Man)	90 (56.2%)	109 (62.3%)	1.025	0.311	1.28^†^
BMI (kg/m²)	25.05 ± 3.16	25.90 ± 3.70	-2.253	0.025	0.246^‡^
Clinical indicators					
Course of disease (years)	5.00 (2.00,10.00)	10.00 (5.00,15.50)	9141	<0.001	0.347^Δ^
Diabetes duration stratified			26.363	<0.001	
≤5	82 (51.2%)	46 (26.3%)			
5-10	50 (31.2%)	63 (36.0%)			
>10	28 (17.5%)	66 (37.7%)			
SBP	135.54 ± 19.30	145.31 ± 23.93	-4.089	<0.001	0.447^‡^
Hypoglycemic regimen			30.145	<0.001	
No medication/diet control	38 (23.8%)	19 (10.9%)			
Oral agents only	90 (56.2%)	75 (42.9%)			
Insulin only	20 (12.5%)	38 (21.7%)			
Oral + insulin	12 (7.5%)	43 (24.6%)			
Hospitalization days	6.0 (5.0,7.0)	7.0 (6.0,9.0)	10116	<0.001	
Inspection indicators					
HbA1c	7.50 (6.70,9.62)	10.00 (8.20,11.30)	7954	<0.001	0.432^Δ^
FPG	7.08 (5.89,8.75)	10.19 (7.50,13.61)	8018	<0.001	0.427^Δ^
TG	1.39 (1.04,1.85)	2.13 (1.36,3.45)	8844	<0.001	0.368^Δ^
TC	4.33 (3.52,5.03)	4.62 (3.98,5.47)	11870	0.016	0.152^Δ^
HDL-C	1.12 (0.97,1.35)	1.00 (0.86,1.21)	17322	<0.001	-0.237^Δ^
LDL-C	2.50 ± 0.91	2.57 ± 1.06	-0.726	0.468	0.079^‡^
ALB	43.90 ± 4.23	41.83 ± 4.85	4.132	<0.001	-0.452^‡^
ALT	19.65 (14.28,28.55)	20.90 (13.95,29.30)	14108	0.903	-0.008^Δ^
AST	21.05 (17.53,26.62)	20.50 (15.80,27.85)	14451	0.611	-0.032^Δ^
BUN	5.78 (4.71,6.93)	6.31 (5.39,8.00)	10946	<0.001	0.218^Δ^
Scr	63.15 (53.70,74.48)	69.10 (57.05,86.95)	11017	<0.001	0.213^Δ^
N	3.08 (2.48,3.92)	4.18 (3.50,5.30)	6854	<0.001	0.510^Δ^
L	1.73 ± 0.60	1.73 ± 0.64	0.090	0.929	-0.010^‡^
PLT	172.60 ± 59.77	200.46 ± 72.36	-3.822	<0.001	0.418^‡^
Comorbidities					
High blood pressure	75 (46.9%)	115 (65.7%)	11.329	<0.001	2.17^†^
Smoking	25 (15.6%)	33 (18.9%)	0.405	0.525	1.25^†^
Drinking	24 (15.0%)	27 (15.4%)	0.000	1.000	1.03^†^
Macrovascular complications					
Coronary heart disease	14 (8.8%)	20 (11.4%)	0.397	0.529	
Cerebrovascular disease	30 (18.8%)	39 (22.3%)	0.441	0.507	
Peripheral vascular disease	57 (35.6%)	105 (60.0%)	18.921	<0.001	
Microvascular complications					
Diabetic retinopathy	1 (0.6%)	66 (37.7%)	Fisher	<0.001	
Diabetic peripheral neuropathy	1 (0.6%)	128 (73.1%)	Fisher	<0.001	

^†^ Proportion, compared using Chi-square test (OR is shown for descriptive purposes only); ^‡^Cohen’s d; ^Δ^Cliff’s delta.

### 3.2 Correlation analysis

Spearman correlation analysis (**Table 2**) revealed a low-to-moderate positive correlation between TyG and lgSII (ρ = 0.27, P<0.001). Both TyG and lgSII showed significant positive correlations with DKD status (ρ = 0.427 and 0.408, respectively, P<0.001), supporting their potential as complementary predictors.

**Table 2:** Spearman correlation coefficients among core predictors and DKD status.

Variable	TyG related	P-value	lgSII related	P-value	Correlation
TyG-lgSII	0.274	<0.001	0.274	<0.001	–
DKD	0.427	<0.001	0.408	<0.001	–
HbA1c	0.475	<0.001	0.238	<0.010	TyG
SBP	0.165	<0.01	0.141	<0.010	TyG
ALB	-0.452	<0.001	-0.077	0.159	TyG
BUN	0.064	0.244	0.150	<0.010	lgSII
Course of disease	0.075	0.172	0.152	<0.010	lgSII
BMI	0.234	<0.001	-0.040	0.461	TyG

### 3.3 Model performance and validation

**Figure 1:**
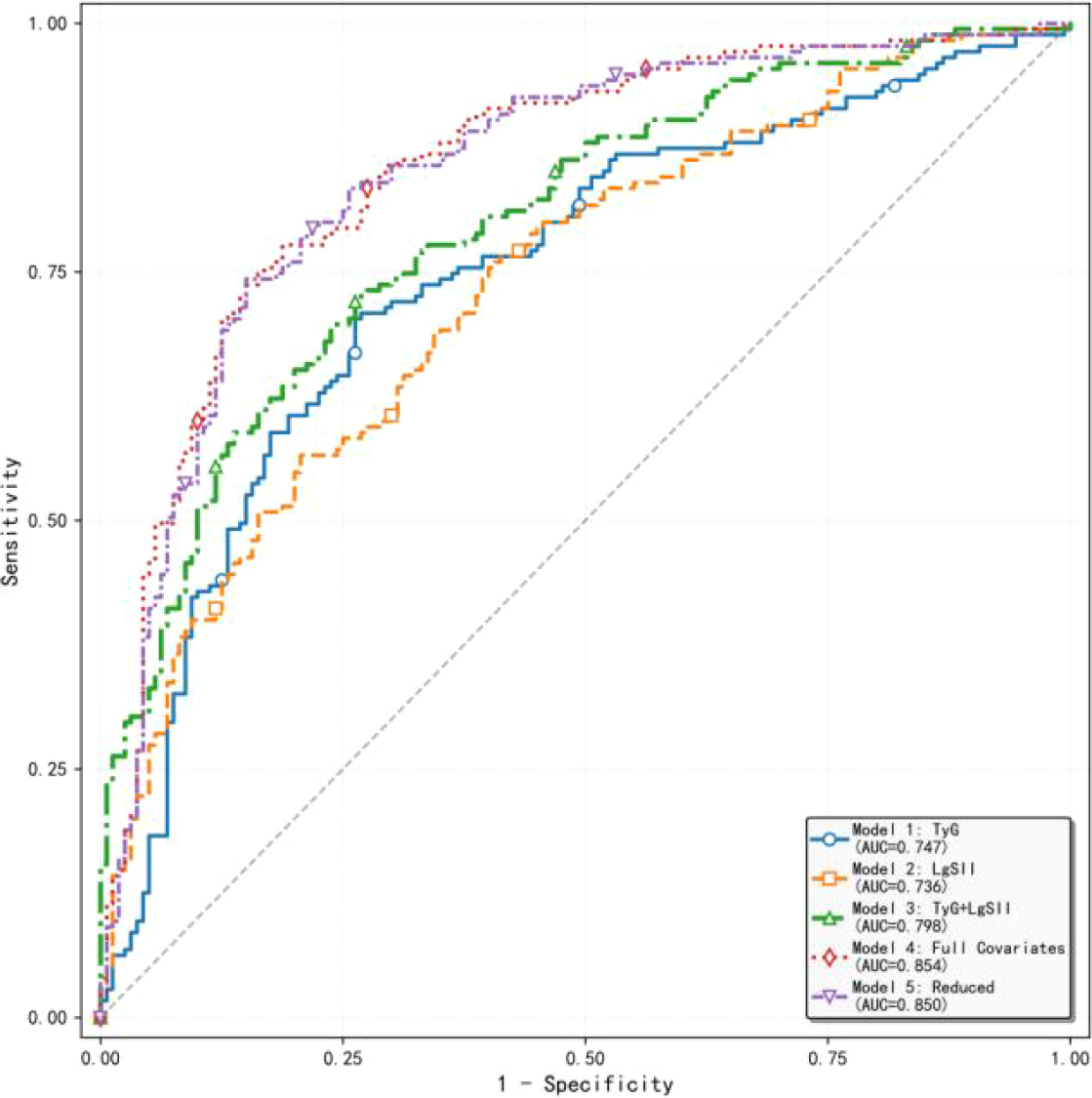
Comparison of ROC curves of the five models.

The streamlined model (Model 5) retained five variables: TyG, lgSII, diabetes duration, SBP, and HbA1c (all VIF <3). Its AUC was 0.850 (95% CI: 0.808-0.891), which was significantly superior to the univariate models (TyG AUC = 0.747, lgSII AUC = 0.736) and the bivariate TyG+lgSII model (AUC = 0.797) (all P<0.001 by DeLong’s test), and not significantly different from the full model (AUC = 0.854, P = 0.32). The optimal probability cut-off from the streamlined model was 0.584, corresponding to TyG >10.03 and lgSII >2.76, with a sensitivity of 74.3% and specificity of 85.0%. The optimal cut-off for lgSII (2.76) corresponds to an SII value of approximately 575.4 on the original scale. Performance metrics for all models are detailed in **Figure 1**, **Tables 3**, **4**.

**Table 3:** Performance comparison of the five prediction models.

Model name	Variables (n)	AUC (95% CI)	Optimal cut-off (Youden)	Sensitivity	Specificity	Youden’s Index	P-value
Model 1 (TyG)	1	0.747(0.690-0.799)	9.358	0.703	0.738	0.440	<0.001
Model2 (lgSII)	1	0.736(0.682-0.787)	2.698	0.566	0.794	0.359	<0.001
Model 3 (TyG+lgSII)	2	0.797(0.745-0.844)	0.507	0.726	0.738	0.463	<0.001
Model 4 (full model)	8	0.854(0.811-0.893)	0.532	0.777	0.813	0.590	0.460
Model 5 (streamlined model)	5	0.850(0.808-0.891)	0.584	0.743	0.850	0.593	

**Table 4:** Multivariable logistic regression analysis of the streamlined model.

Variable	β (voefficient)	OR (95% CI)	P-value	VIF
TyG	0.727	2.07 (1.50-3.12)	<0.001	1.27
lgSII	2.019	7.53 (3.22-18.06)	<0.001	1.14
Course of disease	0.101	1.11 (1.06-1.17)	<0.001	1.03
SBP	0.017	1.02 (1.00-1.03)	0.017	1.04
HbA1c	0.223	1.25 (1.11-1.44)	<0.001	1.25

**Figure 2:**
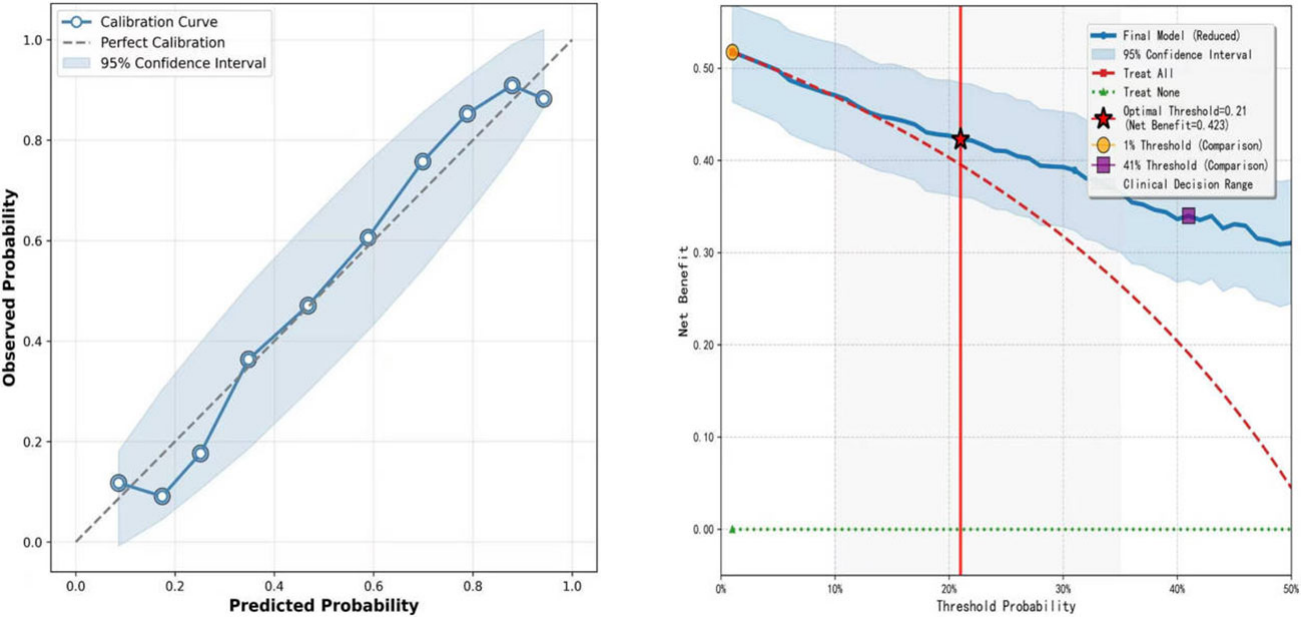
**(A)** Calibration curve of the streamlined prediction model. The dashed line represents the ideal fit. The solid line and points show the observed versus predicted probability of DKD. Hosmer-Lemeshow χ²=10.116, P = 0.257. **(B)** Decision curve analysis for the streamlined model. The y-axis shows the net benefit. The solid black line represents the net benefit of the model across different threshold probabilities. The gray line (“Treat All”) and the horizontal line at y=0 (“Treat None”) are shown for comparison. The model provides a higher net benefit than both strategies within the threshold probability range of approximately 10% to 41%.

The Hosmer-Lemeshow test indicated good calibration (χ²=10.116, P = 0.257). Internal validation demonstrated robust stability: Bootstrap validation (2000 reps) yielded a mean AUC of 0.855 (95% CI: 0.811-0.895), and 5-fold cross-validation produced a mean AUC of 0.844 (SD = 0.047) (**Table 5**). Decision curve analysis (DCA) indicated that the streamlined model provided a superior net benefit compared to the “treat-all” and “treat-none” strategies within a threshold probability range of approximately 10% to 41% (**Figure 2**).

**Table 5:** Internal validation results of the streamlined prediction model.

Validation method	Repetitions	Mean AUC	95% CI	SD	Conclusion
Bootstrap validation	2000	0.855	0.811-0.895	–	stable
5-fold cross-validation	5	0.844	–	SD=0.047	stable

### 3.4 Risk stratification efficacy

Based on the dual cut-offs (TyG>10.03 and lgSII>2.76), risk stratification was highly effective (**Table 6**). The high-risk group constituted 9.3% (31/335) of the total cohort but contained 96.8% (30/31) of DKD patients, corresponding to an OR of 58.62 (P<0.001) compared to the low-risk group. The medium-risk group covered an additional 53.6% of DKD patients. The prevalence gradient across low- (33.9%), medium- (65.7%), and high-risk (96.8%) groups was pronounced.

**Table 6:** DKD prevalence and risk according to TyG and lgSII strata.

Risk group	Criteria	Total patients (n)	DKD patients (n)	Prevalence (%)	Specificity	OR (95% CI)	P-value
Low	TyG ≤ 10.03 and lgSII ≤ 2.76	192	65	33.9	0.350	1	–
Medium	TyG>10.03 or lgSII>2.76	112	80	71.4	0.715	4.88 (3.38-7.56)	<0.001
High	TyG>10.03 and lgSII>2.76	31	30	96.8	0.895	58.62(13.19-70.34)	<0.001

## 4 Discussion

Diabetic kidney disease (DKD), one of the most devastating microvascular complications of type 2 diabetes mellitus (T2DM), exhibits a continuously rising global prevalence and has emerged as a leading cause of end-stage renal disease (ESRD) and cardiovascular mortality (15). Early detection and timely intervention are widely recognized as critical strategies for slowing renal function decline and improving long-term patient outcomes. Current clinical practice for DKD screening primarily relies on the urinary albumin-to-creatinine ratio (UACR) and estimated glomerular filtration rate (eGFR) (16). However, UACR is susceptible to interference from various factors such as infection, physical activity, blood pressure fluctuations, and urine concentration, leading to considerable variability (17). Conversely, eGFR may lack sufficient sensitivity during the early stages of mild renal impairment, and its calculation formulas may introduce bias across populations differing in ethnicity, body habitus, and muscle mass (7). Therefore, developing novel biomarker combinations based on readily available, low-cost, and stable parameters represents a vital research direction for enhancing the efficiency of early DKD identification.

In recent years, metabolic dysregulation and chronic low-grade inflammation have been widely acknowledged as two core pathological mechanisms driving the initiation and progression of DKD (18). The triglyceride-glucose index (TyG), which integrates fasting triglyceride and fasting glucose levels, has become a reliable and straightforward surrogate marker for assessing insulin resistance (IR) (19). Numerous cross-sectional and cohort studies have confirmed that an elevated TyG index is significantly associated with the development of microalbuminuria, decreased eGFR, and a diagnosis of DKD in T2DM patients, with its predictive value being independent of traditional risk factors (13). Concurrently, the systemic immune-inflammation index (SII), a novel composite inflammatory marker derived from complete blood count parameters, offers a more comprehensive depiction of the systemic inflammatory burden by reflecting the balance between neutrophils representing innate immunity and inflammatory activation, platelets involved in inflammation and microthrombosis, and lymphocytes representing adaptive immune status (20). Research indicates that elevated SII is closely linked to an increased risk of carotid atherosclerosis, cardiovascular events, and microvascular complications, including DKD, in T2DM patients (21). Although existing literature has separately explored the associations of TyG or SII with DKD, most studies are limited to evaluating the effect of a single indicator, often with restricted sample sizes, and lack systematic comparison and model development for their combined predictive efficacy within a unified inpatient T2DM population (22). More importantly, how to translate these two indicators, representing the “metabolic axis” and “inflammatory axis” respectively, into a clinically actionable risk stratification tool requires further investigation.

Recent studies have separately explored the predictive value of TyG and SII for DKD. Tu et al. (18) demonstrated that the TyG index was effective for detecting DKD and diabetic peripheral neuropathy in hospitalized T2DM patients (18). Similarly, Wang et al. (20) linked elevated SII to increased cardiovascular and all-cause mortality, suggesting its broader prognostic value (20). However, these studies did not systematically evaluate the additive or synergistic value of combining the ‘metabolic’ (TyG) and ‘inflammatory’ (SII) axes into a single risk stratification model. Our study extends these findings by demonstrating that the combination of TyG and SII significantly improves predictive performance over either index alone.

Addressing these research gaps, this study retrospectively enrolled 335 hospitalized T2DM patients and, for the first time, systematically developed and validated a DKD risk prediction model integrating the TyG index and SII. Our key finding is that a simplified model combining TyG with log10-transformed SII (lgSII), along with diabetes duration, systolic blood pressure, and HbA1c, demonstrated excellent discriminatory ability (AUC = 0.850). This performance was significantly superior to models using TyG alone (AUC = 0.747) or lgSII alone (AUC = 0.736). This aligns with the consensus from prior studies that metabolic disturbances induced by insulin resistance (e.g., hyperglycemia, lipotoxicity) can trigger oxidative stress and endoplasmic reticulum stress, directly damaging glomerular podocytes and renal tubular epithelial cells. Concurrently, the activated chronic inflammatory state, through the release of cytokines such as tumor necrosis factor-alpha (TNF-α) and interleukin-6 (IL-6), further exacerbates endothelial dysfunction, renal fibrosis, and extracellular matrix accumulation, creating a vicious cycle of “metabolism-inflammation” that jointly accelerates DKD progression. Therefore, this study validates that a combined assessment of these dual pathophysiological processes provides a more comprehensive capture of DKD risk, with the improved performance having a solid biological foundation.

The improved performance of combining TyG and SII is biologically plausible. TyG-mediated insulin resistance promotes lipotoxicity, leading to the accumulation of diacylglycerol and ceramides in renal podocytes and tubular cells, which activates protein kinase C (PKC) and induces mitochondrial dysfunction. Simultaneously, chronic hyperglycemia triggers the formation of advanced glycation end-products (AGEs), which bind to their receptor (RAGE) on immune cells (23). This activates the NLRP3 inflammasome, resulting in the cleavage and release of mature IL-1β and IL-18 that drive renal interstitial inflammation and fibrosis. SII, by integrating neutrophil and lymphocyte counts, captures this inflammatory burden. Neutrophil-derived reactive oxygen species (ROS) and neutrophil extracellular traps (NETs) directly damage glomerular endothelium, while reduced lymphocyte counts may reflect an impaired regulatory immune response (24). Thus, TyG and SII together capture a metabo-inflammatory vicious cycle where metabolic stress fuels inflammation, and inflammation exacerbates metabolic dysregulation, jointly accelerating DKD progression.

The study employed a stepwise modeling strategy (from univariate to multivariate) and applied stringent internal validation using Bootstrap resampling (2000 repetitions) and 5-fold cross-validation, ensuring model robustness and reliability. Decision curve analysis (DCA) further demonstrated that within a threshold probability range of 10%-41%, this model provides a superior net clinical benefit compared to “treat-all” or “treat-none” strategies. All variables in the model (TyG, SII, duration, blood pressure, HbA1c) are routinely collected information for hospitalized patients, requiring no additional testing costs, perfectly aligning with the “zero incremental cost” screening concept. The study innovatively proposes a three-tier risk stratification scheme based on dual cut-off values (TyG > 10.03 and lgSII > 2.76). Notably, the high-risk group (both indicators elevated), although comprising only 9.3% of the total cohort, exhibited an extremely high DKD prevalence of 96.8% (OR = 58.62), indicating strong positive predictive value. This offers clinicians, particularly non-nephrology specialists, an intuitive and rapid “red flag” alert tool to help prioritize patients requiring urgent nephrology consultation and intensified management within busy inpatient settings. We explicitly define this model as a screening and triage tool, not a diagnostic tool. It aims to efficiently identify high-risk individuals for DKD from a large pool of hospitalized T2DM patients, thereby guiding them toward confirmatory gold-standard tests (UACR, eGFR) and specialist evaluation, ultimately optimizing healthcare resource allocation and reducing diagnostic delays.

It is important to contextualize our model’s performance against current standard screening methods. While UACR remains the gold-standard biomarker for DKD screening, it is subject to biological variability and may be transiently elevated. In contrast, our TyG-SII model leverages routine metabolic and hematological parameters with higher short-term stability, potentially offering a complementary role (25). Unlike eGFR, which may remain normal until substantial kidney damage has occurred, our model identifies high-risk patients based on systemic metabo-inflammatory dysregulation before overt renal functional decline (26, 27). However, this model is not intended to replace UACR or eGFR but rather to serve as a pre-screening or triage tool to efficiently flag high-risk hospitalized T2DM patients who most urgently require confirmatory UACR/eGFR testing and nephrology referral.

Comparability of the control group. The appropriateness of the non-DKD group as a control population is supported by several dimensions. First, regarding diabetes duration, although the non-DKD group had a significantly shorter median duration (5.0 vs. 10.0 years, P<0.001), this difference reflects the expected time-dependent natural history of DKD as a chronic microvascular complication. Importantly, 48.8% of non-DKD patients had a diabetes duration exceeding 5 years, and 17.5% exceeded 10 years, confirming that this group is not composed of newly diagnosed or mild outpatients. Second, regarding glycemic control, the median HbA1c in the non-DKD group was 7.50% (IQR 6.70-9.62), exceeding the ADA-recommended target of <7.0%, with an upper quartile of 9.62%, objectively confirming substantial glycemic burden. Third, regarding treatment burden, only 23.8% of non-DKD patients received no pharmacologic therapy, while 56.2% required oral agents and 20.0% required insulin (including combination regimens), indicating a real-world clinical management setting. Fourth, regarding complication background, the non-DKD and DKD groups showed comparable prevalence of coronary heart disease (8.8% vs. 11.4%, P = 0.529) and cerebrovascular disease (18.8% vs. 22.3%, P = 0.507), demonstrating shared systemic macrovascular risk profiles. The very low detection rates of diabetic retinopathy and peripheral neuropathy in the non-DKD group (0.6% each) are consistent with DKD being a later-stage microvascular complication and confirm that this group represents hospitalized T2DM patients without overt target organ damage but with genuine metabolic risk. Therefore, the significant differences observed in TyG-SII between groups reflect specific recognition of DKD susceptibility beyond overall diabetes severity.

This study also has several limitations that must be acknowledged. First, as a single-center retrospective study, its level of evidence is inherently lower than that of prospective cohort studies, and it may introduce selection bias. Second, the sample size is relatively modest (n=335), which could affect the precision of risk estimates and limit the power for subgroup analyses, potentially leading to wider confidence intervals for effect estimates in stratified risk groups. Third, and most critically, the lack of external validation on an independent cohort remains a significant barrier to the immediate generalization of our model to other clinical settings. Fourth, although we have corrected the DKD definition according to KDIGO guidelines, the original wording in the protocol could have introduced potential misclassification bias, which we have addressed *post-hoc* by verifying all cases. Fifth, the hospitalized population likely represents a sicker cohort with more advanced disease, limiting generalizability to community or outpatient T2DM populations (spectrum bias). The study did not systematically collect and adjust for detailed medication histories (e.g., metformin, SGLT2 inhibitors, statins) that could significantly influence TyG, SII, and renal outcomes. The cross-sectional design cannot establish whether elevated TyG and SII are causes or consequences of DKD. The model predicts current DKD status, not the future risk of developing DKD. Prospective studies with long-term follow-up and external validation are needed to validate the predictive value of these indicators for DKD incidence and progression.

In conclusion, this study demonstrates that a model combining the TyG index and SII, constructed from routine inpatient laboratory data, is an effective tool for identifying individuals at high risk for DKD among hospitalized T2DM patients. It captures the core “metabolism-inflammation” interplay in DKD pathogenesis and offers significant advantages of low cost, high efficiency, and easy integration. Despite the inherent limitations of a retrospective design, this study provides valuable preliminary evidence for the development of practical clinical decision-support tools.

## 5 Conclusion

In summary, the combination of the TyG index and SII serves as an effective, low-cost, and readily applicable tool for rapid risk stratification of DKD among hospitalized T2DM patients. It leverages routine laboratory data to identify a “metabo-inflammatory” high-risk phenotype with a very high probability of DKD. While not a diagnostic substitute, it holds promise as an efficient triage instrument to optimize resource use and prompt early nephrology assessment, aligning with the goals of preventive medicine and value-based healthcare. The current findings should be considered preliminary evidence, and prospective external validation studies are necessary before this model can be recommended for routine clinical decision-making.

## Data Availability

The original contributions presented in the study are included in the article/supplementary material. Further inquiries can be directed to the corresponding author.
